# Validating the Screening Scale of Pedophilic Crime Scene Behavior

**DOI:** 10.1007/s10508-022-02354-z

**Published:** 2022-07-27

**Authors:** Robert J. B. Lehmann, Sébastien Brouillette-Alarie, Amelie Pedneault, Raymond A. Knight

**Affiliations:** 1 Department of Psychology, MSB Medical School Berlin, Institute of Psychology, Rüdesheimer Str. 50, 14197 Berlin, Germany; 2 École de criminologie, Université de Montréal, Montréal, Québec, Canada; 3 Department of Criminal Justice and Criminology, Washington State University, Pullman, WA USA; 4 Department of Psychology, Brandeis University, Waltham, MA USA

**Keywords:** Pedophilia, Sexual offending, Child pornography, Assessment, Diagnosis, Crime scene analysis

## Abstract

The Screening Scale of Pedophilic Crime Scene Behavior (SSPC) is a seven-item structured rating scale assessing pedophilic sexual arousal. In the current study, we cross-validated the scale’s convergent validity using multiple measures of sexual interest in children (clinical diagnosis of pedophilia, the high fixation/low social competence type of the MTC:CM4, and phallometric assessment of sexual interests toward children) in two independent samples (USA and Canada). In both samples and in relation to all three criteria, the SSPC showed acceptable (phallometry) to excellent (clinical assessment) diagnostic accuracy. Furthermore, the SSPC showed incremental validity in relation to the Screening Scale for Pedophilic Interest and at times outperformed it in convergent validity analyses. The current study also provides psychometric information that can help users choose an appropriate SSPC cutoff score.

## Introduction

Forensic assessment of sex offenders (e.g., risk assessment, diagnosis of paraphilia) is a highly complex process in which multiple psychological and sociological constructs are evaluated. Currently, there is a consensus that sexual deviance and antisocial orientation are the two best categories of predictors for future sexual offending (Hanson & Morton-Bourgon, 2005). Accordingly, forensic practitioners, researchers, and expert witnesses invest considerable resources in the assessment of these constructs. To that end, crime scene data and clues are often used by practitioners to infer what a suspect did during an offense, and, to a larger extent, identify his/her mental, emotional, and personality characteristics (Berg, 2008). According to trait theories of personality (Cattell & Kline, 1977), behavior should reflect personality and, therefore, inform us about risk-relevant psychological constructs (Brouillette-Alarie et al., 2015; Mann et al., 2010). Because behaviors can be fallible indicators of paraphilias (First, 2010), it is important to differentiate specific behavioral indicators of paraphilias from those that could be related to other constructs. In this regard, the current study will validate a diagnostic rating scale assessing such behavioral indicators for pedophilic interest in children within the population of child sexual abusers. Specifically, we will test the convergent validity of the scale in relation to three additional indicators of sexual interest in children. It is important to emphasize that the current study investigates the validity of a forensic tool for people who have offended and does not necessarily have bearing on people with pedophilia in general.

Some risk assessment instruments include crime-scene-related indicators. For example, the Sexual Violence Risk-20 (Boer et al., 1997), a checklist for assessing the risk of sexual recidivism based on structured professional judgment, includes items such as use of weapons, physical harm to victim(s), and high density of sex offenses. In the field of risk assessment, however, meta-analytic research shows considerable variability in the predictive accuracy of crime scene indicators (Gerhold et al., 2007; Hanson & Morton-Bourgon, 2005; McCann & Lussier, 2008), with some making only trivial contributions to prediction (e.g., weapon use, physical force). Therefore, the empirical literature on crime scene behavior indicators needs to be further expanded before such indicators can be reliably used in forensic risk assessment.

In this regard, using multidimensional scaling in a sample of 424 cases of child sexual abuse, Lehmann et al. (2014) identified behavioral themes within crime scene characteristics and linked them with psychological propensities applicable to the sexual abuse of children. Because paraphilias are key motivations for sexual offenses (Seto, 2017), Lehmann and colleagues (2014) also included behavioral indicators of pedophilia and sexual sadism to increase the content validity of the expected themes. Specifically, they included items from the Severe Sexual Sadism Scale (SeSaS; Nitschke et al., 2009) as well as the Screening Scale for Pedophilic Interest (SSPI; Seto & Lalumière, 2001). Because sexual arousal or behaviors indicate that approximately 40–50% of sexual offenders with child victims are not likely to be pedophiles (Seto, 2009), they also added items accounting for alternative explanations of child molestation, such as lack of sexual opportunities with preferred partners and antisociality. They found that crime scene indicators clustered around four latent propensities: fixation/pedophilic interest, sexual aggression/sadism, criminality (e.g., antisociality), and regression (e.g., lack of preferred partner).

They further explored the validity of these four latent propensities. They found that the behavioral theme of fixation/pedophilic interest showed convergent validity with the Static-99 (Hanson & Thornton, 1999) and Static-2002 (Hanson & Thornton, 2003) subscales of sexual deviance and persistence of sexual offending (Lehmann et al., 2014). The two themes representing paraphilia (i.e., fixation/pedophilic interest, sexual aggression/sadism) predicted sexual recidivism, aligning with prior research on sexual offender risk assessment (Nitschke et al., 2009; Seto & Lalumière, 2001). These results were subsequently validated on an independent American sample of sexual offenders against children (Pedneault, 2014), in which offenders with higher fixation/pedophilic interest propensity had elevated sexual recidivism risk. This is consistent with research by Eher et al. (2015) that found that the diagnosis of exclusive pedophilia predicted sexual reoffending. In Pedneault’s (2014) replication sample, the theme of fixation was correlated with the high fixation/low social competence type of the Massachusetts Treatment Center: Child Molester Typology, Version 4 (MTC:CM4; Knight & King, 2012). It is reasonable to conclude from these promising results that crime-scene-related variables may aid in the diagnosis of sexual interests in children, especially because the fixation theme included two of the four variables of the SSPI (i.e., stranger victim, male victim). Because pedophilia has more serious individual consequences (e.g., longer sentences, post-sentence restrictions and requirements) and carries a greater stigmatization than other paraphilias and psychological conditions that are considered risk factors for sexual offending (Lehmann et al., 2020), a valid diagnosis of pedophilia is paramount and efforts to continuously improve existing diagnostic approaches are critical.

### The Screening Scale of Pedophilic Crime Scene Behavior (SSPC)

Based on the research cited above, Dahle et al. (2014) hypothesized that crime scene behaviors beyond SSPI items may improve the accuracy of pedophilic interest assessment. Building on the empirical evidence and on theoretical considerations, they identified 22 variables that cohered into six dimensions of pedophilic crime scene behavior: extant of sexually deviant behavior (e.g., number of offenses), victim selection criteria (e.g., victim gender), offense planning (e.g., luring behavior), violent/controlling offense behavior (e.g., physical force), sexual offense behavior (e.g., anal penetration), and pornography use (i.e., production/possession). Using stepwise regression, they found that seven of the 22 variables showed incremental validity in predicting a clinical diagnosis of pedophilia. These variables were combined to form the Screening Scale of Pedophilic Crime Scene Behavior. In the development sample, the SSPC showed high predictive accuracy for the clinical diagnosis of pedophilia for all offenders who scored 5 or higher (*AUC* = 0.91).

Two of the SSPC items overlap with items from both the SSPI and its latest revision, the SSPI-2 (Seto, Sandler, et al., 2015; Seto, Stephens, et al., 2015). The overlap includes the items age of youngest victim and number of victims. Their operationalization, however, differs in the SSPC. Indeed, the SSPC applies a lower threshold for age of youngest victim (i.e., 9 or younger) than the SSPI and SSPI-2 (11 or younger). This modification ensures that only prepubescent victims are captured by the item, consistent with the criterion for the clinical diagnosis of pedophilia (Blanchard, 2010). For the number of victims item, the SSPI includes offenders having more than one victim under the age of 14 (in the SSPI-2, it is under the age of 15), whereas the SSPC requires two different victims younger than 14 years old. Indeed, the data from the SSPC development sample indicated that two different victims younger than 14 had more discriminatory power than other alternatives. This is also consistent with a phallometric study (Cantor & McPhail, 2015), in which the category of men with three or more child victims contained the smallest proportion of non-pedophiles. The inclusion of the item child sexual exploitation material in both the SSPC and the SSPI-2 is understandable, because research supports child pornography offending as a valid diagnostic indicator of pedophilia (e.g., Seto et al., 2006). The SSPI-2 defines child pornography, however, in accord with the Criminal Code of Canada, in which a child is defined as under the age of 18, whereas the SSPC defines this item according to the German Criminal Code, thereby lowering the age threshold to 14.

The development model of the SSPC included one more item measuring persistence in sexually deviant criminal activity that provided additional discriminatory power, having more than ten separate offenses against one or more victim(s) younger than 14 years old. Also notable is the absence of the male victim item from the SSPC, which is included in the SSPI-2 and was even double weighted in the SSPI. Moreover, Blanchard et al. (2000) found same-sex attraction to be more likely among pedophilic men. A possible explanation for the SSPC results is that the item is a better indicator for gender orientation than for pedophilia in general (Ian McPhail, personal communication, 2019). Finally, the SSPC includes three items that do not overlap theoretically with items included in the SSPI: confidence approach (e.g., Mcalinden, 2006; Olson et al., 2007), anal penetration (e.g., Riggs et al., 2000), and absence of threats to the victim (e.g., Cohen & Galynker, 2002; Hall & Hall, 2007).

### Current Study

The development study suggested that the SSPC is useful for identifying pedophilic interest in offenders and evidenced incremental validity beyond the SSPI. These promising results must, however, be replicated in independent samples to determine their generalizability.^1^ Therefore, the overarching aim of the current study was to cross-validate the SSPC using large, independent samples from different jurisdictions, cultural backgrounds, and time periods. Three specific questions were the foci of this investigation. First, we tried to identify an appropriate SSPC cutoff score for the seven-item scale. Second, we examined the diagnostic accuracy of the SSPC scale using three different diagnostic strategies of sexual interest in children (clinical diagnosis, high fixation/low social competence type of the MTC:CM4, and phallometric assessment data), as multiple positive relations increase the probability that pedophilia has accurately been identified. Third, we tested whether the SSPC adds incremental validity to the SSPI in different samples using different diagnostic strategies.

## Method

### Samples

#### US Sample

The cross-validation sample included 316 offenders with child victims from the USA. These offenders were referred to the Massachusetts Treatment Center (MTC) between 1959 and 1991 for evaluation of their sexual dangerousness, following conviction for at least one sexual offense. Of this sample, 46.2% had been determined to meet the criteria for sexual dangerousness and were committed to the MTC after observation, whereas the remaining were returned to their prior incarceration to complete their sentences. Offenders were included in the current study if they had at least one offense against a victim 14 years or younger and their archival records included full information about the variables necessary to code the SSPI and SSPC. Some of the offenders included had both child and adult victims. At the time of their index offense, offenders were on average 32 years old (*SD* = 11.5); 36% were married; and 72.5% were employed. The individuals included in the study had a mean Static-99 score of 4.2 (*SD* = 2.4), making them a moderate risk sample. Overall, 38.4% of the sample were classified in the high fixation/low social competence MTC:CM4 type (i.e., high pedophilic interest and low adult appropriate sexual/social outlets).

Although the MTC sample was gathered during the first wave of civil commitment legislation in the USA, which began in 1938 in Minnesota, California, Illinois, and Michigan and ended in 1990 in Massachusetts, results from the data on these offenders have been replicated in multiple subsequent studies in both the second wave civil commitment facilities and in generic prisons. The consistent replication of findings suggests that the pedophilia results of the present study should not be distorted by the earlier assessment time of these offenders.

#### Canadian Sample

The Canadian cross-validation sample comes from the Computerized Sex Offender Questionnaire (CSOQ; St-Yves, Proulx, & McKibben, 1994) dataset and included 350 child molesters with only victims younger than 16 years old, as well as 58 mixed offenders, with both minor and adult victims. All offenders received a federal sentence of 2 + years between 1995 and 2000 for at least one hands-on sexual offense. They were evaluated at the Regional Reception Centre in Sainte-Anne-des-Plaines, Québec, Canada, which is a maximum-security penitentiary. Mean offender age at time of evaluation was 41.9 (*SD* = 12.3). Although information on clinical diagnosis was available for all 408 offenders, information on phallometric assessment was available for only 126. Phallometric data were missing for many participants because some did not consent to the evaluation and others did but obtained an invalid profile. There are multiple reasons one can obtain an invalid phallometric profile, the main one being a lack of significant erectile response on any consenting or nonconsenting scenario. According to Michaud and Proulx (2009), more than 30% of phallometric evaluations result in invalid profiles.

### Measures

#### Screening Scale for Pedophilic Interest

(SSPI; Seto & Lalumière, 2001) is a brief structured rating scale designed to assess sexual interest in children among individuals who have committed a sex offense against a child. The scale consists of four items examining the characteristics of child victims: male victim, unrelated victim, 2 + victims, and victim age 11 or younger. Because the item *male victim* is worth two points and the others are worth 1 point, total scores can range from 0 to 5.

#### Screening Scale of Pedophilic Crime Scene Behavior

(SSPC; Dahle et al., 2014) is also a brief structured rating scale designed to assess sexual interest in children among individuals who have committed a sex offense against a child. The scale consists of seven variables with the first four items: (a) number of victims (3 + victims younger than 14), (b) number of offenses (11 + separate offenses against one victim/different victims younger than 14), (c) victim age 9 or younger, (d) child pornography (production or possession) being related to the offense history and the three crime-scene-related items, (e) confidence approach (any indication of trust building), (f) anal penetration, and (g) offender using no threats in the index offense (please confer Lehmann et al., 2018 for tally sheet). Each item is scored dichotomously (*no* = 0, *yes* = 1), and the total score can range from 0 to 7. The interrater agreement ranged from moderate (e.g., confidence approach) to very good (e.g., male victim) with a mean kappa of 0.74.

In the US sample, the seven variables that the SSPC and SSPI comprise were scored by one coder*.* This approach did not raise data validity concerns in light of the descriptive nature of the SSPI and SSPC items, and no issues emerged for the additional nonoverlapping SSPC items that assess offending behavior and involve more judgment. An exact count of the number of offenses against each victim could not, however, always be extracted from archival files, especially in cases involving repetitive assault. Thus, the variable was coded conservatively as “yes” only in cases with positive evidence supporting that more than 10 offenses were committed against victims younger than 14. In addition, the time period of the data collection (i.e., 1959–1990) warrants a note. Child pornography was less accessible in these years, and such pornography use was not consistently examined during investigations, especially for offenders evaluated at MTC during the earliest years.

In the Canadian sample, the SSPC was scored retroactively by recoding existing variables in the CSOQ dataset. The recoding syntax was approved by lead SSPC authors. Even though this implies that SSPC interrater reliability data were not available for the Canadian sample, interrater reliability data about the CSOQ exist and indicate almost-perfect agreement (Brouillette-Alarie & Proulx, 2019). There was insufficient information in the CSOQ to score one item (child pornography). Fortunately, the SSPC can be scored without this item. Total scores therefore ranged from 0 to 6 in the Canadian sample.

### Criterion Validity

According to Seto (2018), evidenced-based clinical assessment should rely on self-report, behavioral history, and phallometric testing of sexual arousal patterns. Therefore, multiple criteria were used to examine the concurrent validity of the SSPC.

#### Clinical Diagnosis

Pedophilic disorder diagnoses rely on patients’ self-reports about sexual interests in children and their persistence, recurrence, intensity, and duration. Note that in the Diagnostic and Statistical Manual of Mental Disorders—5th edition (DSM-5; American Psychiatric Association, 2013), one can diagnose pedophilic disorder using only client behavior. Clinical diagnoses were available for the Canadian sample. In the Canadian sample, an offender was considered to have pedophilic disorder if the participant was diagnosed with pedophilia by a mental health expert at least once in his life. This information was obtained from the participant’s clinical records.

#### Phallometric Assessment

Phallometric assessment is a psychophysiological assessment method frequently used in forensic settings to identify deviant and non-deviant sexual interests by measuring changes in penile circumference or blood volume in the presence of stimuli depicting individuals of different ages, sexes, and involved in varying sexual activities. Phallometric stimuli vary considerably. Audio recordings describing sexual scenarios, visual depictions of nude or clothed subjects, and videotapes or computer-generated images have all been used (McPhail et al., 2017). In the current study, only the Canadian sample included data on penile plethysmography (PPG). PPG data were measured with penile circumference using a mercury-in-rubber strain gauge. Participants were exposed to a French version of Quinsey and Chaplin’s (1988) audio scenarios (see Quinsey & Chaplin, 1988 and Barsetti et al., 1998 for validation data). These scenarios comprised six situations: neutral scenario (control), consensual sex with an adult men/women (non-deviant), noncoercive sexual contacts with a prepubescent boy/girl (deviant), coercive sexual contacts with a prepubescent boy/girl (deviant), rape (full sexual intercourse) of a prepubescent boy/girl (deviant), and nonsexual violence toward a prepubescent boy/girl (deviant). As mentioned earlier, only a subsample of Canadian offenders had a valid phallometric profile (*n* = 126). PPG indices were computed using *Z*-score difference indices, which have been found to maximize discriminant validity among sexual aggressors of women, children, and non-criminalized community members (Harris et al., 1992). For each offender, an overall *Z*-score pedophilic deviance index was computed, indicating the difference in *Z* scores between the highest response to deviant pedophilic stimuli minus the highest response to non-deviant stimuli. Consistent with evidence that pedophilic sexual preference is distributed as a distinct taxonic (Brankley et al., 2022; McPhail et al., 2018; Schmidt et al., 2013) and in accord with prior cutoffs (Seto, Sandler, et al., 2015; Seto, Stephens, et al., 2015), we used a cutoff of *Z* = 0.25 (indicating a 0.25 *SD* higher response for deviant stimuli compared to non-deviant stimuli) to determine the presence of pedophilic preferences as measured by PPG.

#### MTC:CM4

Early typological work on sexual offenders of children has always included a category seemingly capturing offenders with pedophilic interest (e.g., the Fixated type in MTC:CM1, Knight, 1988). The MTC:CM taxonomic work constitutes a thrice-revised and empirically validated classification of sex offenders against children based first on a sample of offenders referred to MTC, but including in later revisions multiple convicted sex offenders in both generic prisons and other civil commitment centers (Knight & King, 2012). The most recent revision of the typology has the added advantage of mapping etiological mechanisms unto the typology itself, thereby merging two bodies of research from differing perspectives (Knight & King, 2012). Of note, the high fixation/low social competence type of the MTC:CM4 comprises an apparently discrete cluster of offenders who share signs of neurodevelopmental perturbations, plausibly inferred from a history of pregnancy and birth complications and poorer cognitive functioning including higher prevalence of low intelligence, mental retardation, and attention deficits (Knight & King, 2012). These neurodevelopmental indicators converge with increased sexual preference for children in offenders in the MTC:CM4 type. Knight and King argued that this convergence supports the neurodevelopmental hypothesis of pedophilia, according to which in utero and early postnatal risk factors are linked with pedophilic interest (e.g., Blanchard et al., 2002). Information on the MTC:CM4 classification of offenders was available for the US sample and was used as one of the three variables measuring pedophilic interest. Knight et al. (1989) reported good reliability for the fixation discrimination variable (Cohen’s *κ* = 0.67) and excellent reliability for the social competence judgment variable (Cohen’s *κ* = 0.84).

### Plan of Analysis

We modeled our analyses following Dahle et al. (2014). Because the purpose of the current paper is cross-validation, we included the findings of Dahle et al. (2014) for comparison purposes when appropriate. Because the SSPC was published before the SSPI-2, and Dahle et al. (2014) compared the SSPC to the SSPI, we will likewise only test the incremental validity of SSPC to SSPI.

#### Cutoff Score

To determine an appropriate cutoff score, we investigated the proportion of offenders identified as pedophilic in relation to the three criteria mentioned above for each value of the SSPC. We expected the likelihood of being identified as pedophilic to increase with SSPC scores, independent of the criterion. Previous analyses by Dahle et al. (2014) suggested a cutoff score of 5.

#### Discrimination

The ability of the SSPI and SSPC scales to discriminate between offenders with and without a clinical diagnosis of pedophilia, as well as offenders classified in MTC:CM4 high fixation/low social competence type or identified as having pedophilic preferences by our PPG index, was assessed by the area under the curve (AUC) from receiver operating characteristic (ROC) analysis (e.g., Rice & Harris, 1995). As a general rule, Hosmer et al. (2013) consider AUC values between 0.7 and 0.8 as acceptable discrimination, between 0.8 and 0.9 as excellent discrimination, and larger than 0.9 as outstanding discrimination. AUCs are considered statistically significant if their confidence intervals do not include 0.50.

#### Incremental Validity

Incremental validity assesses whether a new measure adds to the prediction of a criterion (e.g., diagnosis of pedophilia) beyond existing measures in the model. Additional measures may add incrementally by either improving the measurement of constructs already included or by assessing a new criterion-related construct. Because already existing diagnostic scales have become entrenched in clinical practice (e.g., SSPI), the burden of proof for the developers of new scales has shifted to demonstrating that their scale provides incremental validity beyond those already in use (Hunsley & Meyer, 2003). Accordingly, we were interested in determining whether the newly developed SSPC would increase the diagnostic accuracy of pedophilia beyond the SSPI. To test the incremental validity of the SSPC, both scales (predictors) were entered sequentially into three hierarchical logistic regression models, each using a different pedophilia criterion as the dependent variable: (a) clinical diagnosis of pedophilic disorder; (b) PPG-measured index of sexual interests toward children (deviance index of 0.25 or more); and (c) the high fixation/low social competence MTC:CM4 type.

## Results

### Cutoff Score

The average SSPC score in the US sample was 2.48 (*SD* = 1.21, range 0–6) and in the Canadian sample 2.05 (*SD* = 1.29, range 0–6). The proportion of offenders identified as pedophilic are presented in Table 1. Table 1 also includes the German development sample as a reference. The distribution of SSPC scores in relation to clinical diagnoses indicates that, like the German developmental sample, 100% of the offenders in the Canadian sample who scored 5 or higher on the SSPC had a clinical diagnosis of pedophilic disorder. For the US sample, those who had a score of 6 or more (*n* = 2) were all diagnosed with pedophilic disorder as measured by the MTC:CM4 type. Among those having 5 or more on the SSPC, 66.7% were in the MTC:CM4 type. Whereas none of the 126 offenders in the Canadian PPG subsample scored 6 or 7 on the SSPC, 83.3% of the offenders scoring 5 on the SSPC were identified as pedophilic according to the > 0.25 PPG pedophilia preference cutoff.

**Table 1:** Proportion of Offenders Identified as Pedophilic in Relation to Different Criteria

SSPC score	Dahle et al. (2014)		Canadian sample		US sample		Canadian sample	
	*N*	Clinical diagnosis (%)	*N*	Clinical diagnosis (%)	*N*	MTC:CM4 type (%)	*N*	PPG cutoff = 0.25 (%)
0	0	–	56	12.5	11	–	6	33.3
1	12	0.0	81	25.9	52	22.7	17	35.3
2	25	16.0	111	64.0	113	35.6	41	46.3
3	25	12.0	116	82.8	73	48.6	46	60.9
4	22	63.6	31	93.5	49	60.4	12	75.0
5	12	100.0	11	100.0	16	62.5	6	83.3
6	14	100.0	2	100.0	2	100.0	–	–
7	3	100.0	–	–	0	–	–	–

*SSPC* Screening scale of pedophilic crime scene behavior

### Criterion Validity/Classification Accuracy

Given that there is no gold standard for diagnosing pedophilia, the current study considered three different diagnostic strategies, that is, clinical diagnosis, MTC:CM4 type, and PPG, to examine the criterion validity of the SSPC. For each method, AUCs were computed to examine the classification accuracy of the SSPC in identifying cases with pedophilia. The SSPI was also added as a comparison measure (Table 2).

**Table 2:** Diagnostic Accuracy of the SSPC and SSPI

	Clinical diagnosis		MTC:CM4 type	PPG cutoff = 0.25
	Dahle et al. (2014) (*n* = 113)	Canadian sample (*n* = 408)	US sample (*n* = 316)	Canadian sample (*n* = 126)
	AUC (95% CI)	AUC (95% CI)	AUC (95% CI)	AUC (95% CI)
SSPC	0.91* (0.86–0.97)	0.82* (0.78–0.86)	0.71* (0.65–0.77)	0.65* (0.55–0.74)
SSPI	0.71* (0.63–0.79)	0.74* (0.69–0.79)	0.77* (0.72–0.83)	0.65* (0.55–0.75)

*SSPC* Screening scale of pedophilic crime scene behavior, *SSPI* Screening scale for pedophilic interests

*p < .05. AUCs are considered statistically significant if their confidence intervals do not include 0.50

First, for clinical diagnoses of pedophilic disorder the AUC indicated that the SSPC was able to reach excellent discrimination in the Canadian cross-validation sample (Table 2). Table 2 also includes the German development sample as a reference. In comparison, the SSPI showed only acceptable discrimination in the Canadian cross-validation sample. Second, the criterion validity was assessed between the MTC:CM4 high fixated/low social competence type and the SSPC and SSPI. Both the SSPC and SSPI scales showed acceptable discrimination. Third, SSPC and SSPI scores were both below Hosmer et al.’s (2013) *AUC* = 0.7 criterion for acceptable diagnostic accuracy for the PPG measures in the Canadian sample, although both scales yielded statistically significant predictions. Overall, results indicate that both the SSPC and SSPI scales had meaningful relations with three different criteria of sexual interest in children, with the SSPC slightly outperforming the SSPI.

### Incremental Validity

Hierarchical block-wise logistic regressions using backward LR were calculated to test incremental validity of the SSPC. For each regression analysis, the SSPI was entered into the first block of the model, and the SSPC was entered in a second block to test whether the SSPC added incremental validity to the SSPI (Table 3). These analyses were followed by reversing the variable order.

**Table 3:** Incremental Validity of the SSPC in relation to the SSPI

	Clinical diagnosis		MTC:CM4 type		PPG cutoff = 0.25	
	Canadian sample		US sample		Canadian sample	
	B (*SE*)	Exp(*b*)	B (*SE*)	Exp(*b*)	B (*SE*)	Exp(*b*)
Model 1						
Step 1						
SSPI	0.61 (0.08)***	1.84	0.81 (0.11)***	2.25	0.35(0.13)**	1.42
Step 2						
SSPI	0.24 (0.10)*	1.27	0.73 (0.11)***	2.08	0.23 (0.15)	1.26
SSPC	1.11 (.14)***	3.03	0.25 (0.12)*	1.29	0.36 (0.20)	1.43

SSPC, Screening scale of pedophilic crime scene behavior; SSPI, Screening scale for pedophilic interests; MTC:CM4 type, Massachusetts treatment center: child molester typology,;version 4, PPG, Penile plethysmography

*p < .05, **p < .01, ***p < .001

Like the German development sample, in the Canadian cross-validation sample SSPC added incrementally to the SSPI in predicting clinical diagnoses of pedophilia. When the SSPI was entered at Block 1, there was a significant effect (*χ*^2^(1) = 72.39, *p* < 0.001) explaining 21.9% (Nagelkerke) of the variance in the dependent variable. Entering the SSPC at Block 2 showed a significant effect for this block (*χ*^2^(1) = 85.17, *p* < 0.001) explaining 43.1% (Nagelkerke) of the variance. This equals a significant 21.2% change in R-squared for the added block (change -2 log-likelihood = 85.17, *p* < 0.000). Reversing variable order led to a significant Block 1 effect (*χ*^2^(1) = 151.27, *p* < 0.001) for the SSPC, explaining 41.7% (Nagelkerke) of the variance. Adding the SSPI led to a significant Block 2 (*χ*^2^(1) = 6.29, *p* = 0.012), again explaining 43.1% of the variance (Nagelkerke). This equals a significant 1.4% change in R-squared for the added block (change -2 log-likelihood = 6.29, *p* = 0.012).

When using the MTC:CM4 type, the logistic regression model also found both scales were significant predictors, with the SSPI showing a larger effect. Entering the SSPI in the first Block yielded a significant effect (*χ*^2^(1) = 73.08, *p* < 0.001), explaining 29.7% (Nagelkerke) of the variance. Adding the SSPC in the second block also showed a smaller but significant effect (*χ*^2^(1) = 4.48, *p* = 0.034), corresponding to an explanation of 31.3% (Nagelkerke) of the variance. This equals a significant 1.6% change in R-squared for the added block (change -2 log-likelihood = 4.48, *p* < 0.05). When the order was reversed, and the SSPC was entered in the first block, a significant effect was found (*χ*^2^(1) = 28.18, *p* < 0.001), explaining 12.3% (Nagelkerke) of the variance. Adding the SSPI in the second block also yielded a significant effect (*χ*^2^(1) = 48.37, *p* < 0.001), explaining 31.3% (Nagelkerke) of the variance. This equals a significant 19.0% change in R-squared for the added block (change -2 log-likelihood = 48.37, *p* < 0.001).

In the final model using PPG data, both the SSPI and the SSPC were found to be significant predictors, but neither showed incremental validity. When the SSPI was entered in Block 1 and SSPC was entered in Block 2 of a hierarchical logistic regression analysis, there was significant effect in Block 1 (*χ*^2^(1) = 7.97, *p* = 0.005), explaining 8.2% of the variance (Nagelkerke), but only a marginally significant effect for Block 2 (*χ*^2^(1) = 3.32, *p* = 0.068) explaining 11.5% of the variance (Nagelkerke). This equals a non-significant 3.3% change in R-squared for the added block (change -2 log-likelihood = 3.32, *p* = 0.068). Reversing the order of variables revealed a significant effect in Block 1 (*χ*^2^(1) = 8.77, *p* = 0.003) explaining 9.0% of the variance (Nagelkerke), but no significant effect for Block 2 (*χ*^2^(1) = 2.52, *p* = 0.113).

## Discussion

The objectives of the current study were to validate the SSPC, a scale based on offense behavior history to identify pedophilic interest, determine its cutoff score, and test its incremental validity beyond a similar existing measure (the SSPI). We presented data from two different countries and three different criteria measuring the relevant construct (i.e., sexual interest in children).

### Screening Scale of Pedophilic Crime Scene Behavior Cutoff for Determining Pedophilic Interests

Like the development sample the two cross-validation samples produced a steady increase across all three diagnostic strategies in the proportion of offenders identified as pedophilic as the SSPC score increased.^2^ Consistent with the German development sample, all offenders in the Canadian cross-validation sample who scored 5 or higher received a diagnosis of pedophilia. In the US sample, more than 60% of offenders scoring 5 on the SSPC were part of the high fixation/low social competence MTC:CM4 type, and all offenders scoring 6 were diagnosed in the type. Note that the MTC:CM4 qualification describes mostly offenders with preferential sexual interest toward children that are likely to have multiple male prepubescent victims, akin to the fixated child molester of Groth et al. (1982). Therefore, it may contain a more “severe” substrata of offenders with pedophilic disorder, as the DSM diagnosis can be given to offenders with preferential *and* nonpreferential interests toward children (Groth et al., 1982). Thus, it is not surprising that the SSPC cutoff emerging from the MTC:CM4 sample was slightly higher than those indicated by the clinical diagnoses samples. For the criterion of having Z-scores > 0.25 on PPG pedophilic scenarios, 83.3% of offenders with a score of 5 on the SSPC were classified as pedophilic. This finding is comparable to prior results (Seto, Sandler, et al., 2015; Seto, Stephens, et al., 2015), in which 73% of offenders reaching a score of 5 on the SSPI-2 were pedophilic. Taken together, results from the three samples suggest that a score of 5 might serve as a usable cutoff for the diagnosis of pedophilia based on SSPC assessment. Although this cutoff is reasonable, given the distribution of scores against diagnosis, MTC typological assignment, and phallometric cutoff score, it focuses on limiting the number of false positives, and future studies should further investigate this point.

### Criterion Validity of the Screening Scale of Pedophilic Crime Scene Behavior

Psychological assessment research should require criterion measures that have support for good reliability and validity. To date, there is no gold standard for the actual diagnosis of pedophilia. Indeed, some data indicate that up to one in three clinical diagnoses of pedophilia may be wrong (Mokros et al., 2018). Even though psychological diagnoses are imperfect, Dahle et al. (2014) used clinical diagnosis as the only criterion to develop the SSPC. To overcome this criterion limitation, the current study examined agreement across alternative diagnostic strategies of pedophilic interests, namely PPG indices and the MTC:CM4 typology. First, consistent with the high AUC with clinical diagnoses of pedophilic disorder in the German developmental sample, excellent diagnostic discrimination for pedophilia emerged in the Canadian cross-validation sample. In comparison, the SSPI showed only acceptable discrimination in both samples. Second, both the SSPC and SSPI showed acceptable discrimination in differentiating sexual offenders against children who were and were not in the high fixated/low social competence types of the MTC:CM4. Third, SSPC and SSPI scores were both below acceptable diagnostic accuracy for the PPG measures of the Canadian sample, although both scales performed better than chance. It is important here to note that the problem may not lie in the SSPC, but rather in PPG measures. Indeed, in a study similar to the one undertaken here, Longpré et al. (2016) tested the convergent validity of three measures of sexual sadism: DSM diagnoses, the SeSaS, and PPG measures. PPG measures were found not to converge with the other two measures, which did fit together. Similarly, a study with 130 child sexual abusers examining different diagnostic methods found no significant relation between DSM-IV-TR pedophilia diagnosis and phallometric test results (Wilson et al., 2011). Seto et al.’s (2016) study of 79 participants (contact sexual offenders, child pornography offenders, concerns about possible sexual interest in children) suggested, however, that the DSM-IV-TR criteria show criterion-related validity for self-report and for objective measures of sexual interest in children. The reliability and validity of PPG measures are known to be notoriously low, with only the discriminant validity of pedophilic scenarios being acceptable (Marshall & Fernandez, 2003). Therefore, SSPC’s poor convergence with PPG measures constitutes at best a weak disconfirmation.

Overall, both the SSPC and SSPI had meaningful relations with three different measures of sexual interest in children, with the SSPC slightly outperforming the SSPI in most cases. Thus, results from the current study support the SSPC as a useful measure of pedophilic sexual interest. Because the DSM-5 diagnostic criteria for pedophilia include behavioral indicators, using the SSPC offers a valid alternative to assessing pedophilic interest by coding a number of easily accessible criminal history and crime characteristic variables. Research generally shows better agreement for structured assessment than for clinical diagnoses (Zimmerman, 1994).

### Incremental Validity of the Screening Scale of Pedophilic Crime Scene Behavior

Because the SSPC is modeled after the SSPI, it is essential to test the incremental validity of the new measure. Based on the results of Lehmann et al. (2014), it seemed reasonable to assume that behavioral crime scene variables along with victim characteristics could be proxy measures of pedophilic interests. The current study also suggests that the SSPC may have explanatory power beyond that of the SSPI. If they assessed strictly the same constructs, SSPC would not have added significant predictive variance to the SSPI. Except for PPG data, SSPC added incremental validity to the SSPI in all three samples (including the development sample). For clinical diagnosis, in both the German and Canadian samples, the SSPC was the measure that most contributed to prediction. Because the SSPC was developed in the German sample, cross-validating the results with an independent sample was necessary to avoid overfitting the data. In the US sample’s MTC:CM4 analyses, the SSPI contributed more to prediction, echoing the AUC analyses. These incremental validity findings indicate that the SSPC assesses criterion-relevant variance that is not considered in the SSPI. The additional variance covered could be explained by the three additional items assessing whether the modus operandi was driven by paraphilic interests (gaining and maintaining sexual access to a child).

For example, the *confidence approach* item, which includes befriending, grooming, luring, and giving goodies to the victim, could be an important component of pedophilia, whereas *using threats to harm the victim* would not. The *confidence approach* item also seems to assess explicit planning and well-crafted strategies that result in a sexual offense, as discussed in the approach-explicit pathway of Ward and Hudson (1998). The *anal penetration* item might capture some of the variance in the *male victim* item (e.g., boy-oriented pedophilia), but it could also tap into other constructs, for example, strategies that result in establishing a degree of control that makes anal penetration possible. These hypotheses suggest that other behavioral indicators of pedophilia could improve the classification accuracy of the SSPI, as Seto, Sandler, et al. (2015), Seto, Stephens, et al. (2015)) have suggested.

Importantly, the three new SSPC items share the advantages of SSPI items: They are readily available from official records, not dependent on self-report, and relatively easy to score (Seto, Sandler, et al., 2015; Seto, Stephens, et al., 2015). Thus, the SSPC should not entail significant additional costs in comparison to the SSPI or SSPI-2 (e.g., additional time to score, lower reliability, or missing information). Also, applying more stringent thresholds for age (i.e., younger) and number of victims (i.e., more) might make the SSPC a more precise measure of pedophilia than the SSPI-2. Indeed, SSPI’s authors themselves conclude that their scale may better reflect pedohebephilia than pedophilia (Stephens et al., 2019). Because this measure is coded from official records of crime scene behavior as well as offending behavior, it might be more appropriate to refer to this measure as Screening Scale of Pedophilic Offense Behavior.

Finally, Seto, Sandler, et al. (2015), Seto, Stephens, et al. (2015)) pointed out that offense history might not accurately reflect pedophilic interests for individuals who have not had sufficient opportunity or time to offend. For these individuals, the SSPC might be a superior measure, because it puts more emphasis on modus operandi and less on criminal repetition.

### Limitations

The current study is not without limitations. First, not all samples included the full range of SSPC scores because analyses relied on archival data (e.g., the Canadian sample had no data on child pornography, and a portion of the US sample was too old to have witnessed the increased availability of online child and adult pornography). Indeed, although the US sample comprises offenders assessed between 1959 and 1991, the analyses indicated that results hold up across different time periods. Nevertheless, future studies should aim to include more current samples. Second, as mentioned above, for the Canadian and US samples SSPC scoring relied on recoding of variables already present in databases put together for other projects. It is therefore possible that not all intricacies of the SSPC scoring manual were respected, due to the nature of the data. Third, because the PPG data were based on a small subsample of the Canadian database, the reduced power of PPG-related analyses may have limited discrimination. Coincidentally, the presence of phallometric data for some Canadian offenders was unlikely to be randomly distributed: Some refused to take part in the test and others obtained invalid phallometric profiles. According to Castonguay et al. (1993), penile response magnitude in phallometric assessment is associated with multiple factors such as age, judiciary status, and offense characteristics. Therefore, results of the current paper based on phallometric data must be interpreted in light of sampling considerations. Fourth, even though the three nonoverlapping SSPC items share the advantages of SSPI items, they also involve more judgment and thus may take a little more time to complete and incur some costs in lowering interrater reliability. Furthermore, not all data included in a file are always recorded in a useful way for SSPC rating purposes, as was the exact count of offenses per victim in the US sample. Fifth, all three criteria for pedophilia were never present in both datasets. The pedophilia diagnosis and PPG data were present in the Canadian sample, but not in the US sample, and the MTC:CM4 data were present in the US sample and not in the Canadian sample. Future studies should try to gather data on all three criteria in one dataset and add diagnostic indicators such as self-report and indirect measures (e.g., visual reaction time).

### Conclusion

In sum, the current study indicated that, consistent with analyses calculated on the German developmental sample, the SSPC appeared useful in two independent cross-validation samples as a structured measure of pedophilic interests in forensic samples. The probative value of the new scale is supported by the reliable covariation of three different diagnostic strategies (i.e., clinical diagnosis, MTC:CM4 type, and PPG data). Moreover, the new items of the SSPC added incrementally to the criterion-related validity of the SSPI. They also integrate an important aspect of sex offenders’ modern lives, which is the impact of illegal pornography in their index offending process (although the SSPI-2 added that aspect to the original SSPI). Finally, as its authors suggest (Stephens et al., 2019), the SSPI-2 may be a measure of pedohebephilia, whereas the SSPC targets pedophilia only. Thus, the SSPC may offer a more specific measure of pedophilia that is relevant in multiple forensic contexts.

## Footnotes

1 The German development sample will be referenced as a comparison because the results have not been published in English language.

2 Note one exception emerged in the development sample in which there was a slight decrease in pedophilic diagnosis from 2 to 3.
